# From Toxic Ingestion to Cancer: Dramatic Acidosis as a Myeloma-Defining Event

**DOI:** 10.7759/cureus.38542

**Published:** 2023-05-04

**Authors:** Nicholas J Burkholder, Lauren M Sweet, Erin L Kelly

**Affiliations:** 1 Internal Medicine, Brooke Army Medical Center, San Antonio, USA; 2 Critical Care, University of Rochester, Rochester, USA

**Keywords:** altered mental status, anion gap metabolic acidosis, multiple myeloma, acute kidney failure, osmolar gap

## Abstract

Acute kidney failure has myriad causes and presentations. This is a case of an individual with a history of alcohol abuse and a previous suicide attempt presenting with acute kidney failure and altered mentation accompanied by an anion gap metabolic acidosis with an elevated osmolar gap. These findings were concerning for toxic alcohol ingestion, but the patient was ultimately diagnosed with multiple myeloma. This case demonstrates the multiple factors that can impact both the anion and osmolar gaps. It shows that the traditionally held dogma about the meaning of anion or osmolar gaps may cloud an otherwise more obscure etiology. It illustrates a dramatic presentation of acute myeloma, for which early recognition is essential to initiate appropriate chemotherapy for a chance at preservation of renal function.

## Introduction

Acute kidney failure can present in a myriad of forms, and detailed history taking is a crucial piece in determining the etiology and guiding treatment. This can be challenging, particularly when renal failure is accompanied by altered mentation. A wide differential and attention to ancillary labs should be considered throughout [1]. Even with reliable first-hand collateral information, the true etiology can remain elusive, as was in this case of multiple myeloma-induced acute renal failure. This article was previously presented at the nephrology breakout session at the 2022 Tri-Service ACP Conference on September 8, 2022.

## Case presentation

A 62-year-old male with a history of alcohol abuse, bipolar disorder on lithium, and a previous suicide attempt via metoprolol overdose and wrist-cutting presented to the emergency department with altered mental status, hypothermia to 35.5°C (96 °F), and mild hypertension after his wife found him lying outside for between eight and 12 hours.

In the emergency department, his labs were remarkable for metabolic acidosis with a pH of 7.18, serum bicarbonate of 6 mmol/L, lactate of 5 mmol/L, and an anion gap of 26. His serum creatinine was 39.6 mg/dL and blood urea nitrogen (BUN) 240 mg/dL, both values previously normal. His potassium was also elevated at 6.2 mmol/L. Urinalysis revealed 2+ protein and blood with a urine pH of 5.0 and specific gravity of 1.015. He had only mild macrocytic anemia with a slight neutrophilic leukocytosis (Table 1), corrected calcium of 10.7 mg/dL, and normal liver enzymes.

**Table 1:** Admission Labs

Lab Test	Patient’s Presenting Values	Reference Range
Serum pH	7.18	7.35-7.45
Serum Sodium	142 mmol/L	135-145 mmol/L
Serum Potassium	6.2 mmol/L	3.5-5.1 mmol/L
Serum Chloride	110 mmol/L	94-106 mmol/L
Serum bicarbonate	6 mmol/L	20-29 mmol/L
Anion Gap	26 mmol/L	<12 mmol/L
Osmolar Gap	42	10-15
Serum Creatinine	39.6 mg/dL	0.60-1.30 mg/dL
Blood Urea Nitrogen	240 mg/dL	7-25 mg/dL
Serum Calcium	10.7 mg/dL	8.2-10.3 mg/dL
Serum Albumin	4.2 g/dL	3.2-5.0 g/dL
White Blood Cells	10.62 K/mcL	3.40-10.40 K/mcL
Absolute Neutrophil Count	9.59 K/mcL	1.50-6.60 K/mcL
Hemoglobin	12.3 g/dL	12.8-17.1 g/dL
Lactate	5 mmol/L	0.5 mmol/L
Lithium	0.6 mmol/L	0.5-1.0 mmol/L
Erythrocyte Sedimentation Rate	109 mm/hr	3-46 mm/hr
C-Reactive Protein	16.16 mg/L	0.00-10.00 mg/L
Urine pH	5.0	5.0-7.5
Urine Specific Gravity	1.015	1.001-1.035
Urine Blood	2+	Negative
Urine Protein	2+	Negative

Most notably, the patient had an osmolar gap that was elevated to 42 mOsm/L (normal <10 mOsm/L), which, in the context of his history of alcoholism and previous suicide attempt, raised concerns for acute kidney failure secondary to toxic ingestion. Medical toxicology and nephrology were consulted and methanol, ethylene glycol, and propylene glycol levels were sent. The patient was started on emergent hemodialysis (HD), treated with fomepizole, and admitted to the medical intensive care unit (MICU). Beta-hydroxybutyrate was elevated to 9.1 mmol/L, however, a full toxicology screen eventually resulted negative or within normal limits, including his lithium level.

Over the following three sessions of daily HD, his encephalopathy improved, followed by his acidemia. By the end of his third HD session, his osmolar gap closed. Once lucid, the patient admitted to self-discontinuing his lithium and other psychiatric medications weeks earlier but staunchly denied any suicidal ideation, including toxic ingestion. Concerningly, he remained oliguric. As other explanations for his presentation and lack of renal recovery were systematically ruled out, further labs and a renal biopsy were obtained.

Upon return, renal pathology showed kappa light chain cast nephropathy and tubular necrosis with oxalate crystals within the tubules. Around this time, serum and urine protein electrophoresis results indicated an immunoglobulin A (IgA) kappa monoclonal gammopathy with a free light-chain (FLC) ratio of 419. With the underlying cause now clear, hematology was consulted for rapid initiation of therapeutic plasmapheresis to reduce free light chain burden. Bone marrow biopsy confirmed plasma cell myeloma with t(11;14) translocation and the patient was started on chemotherapy with cyclophosphamide, bortezomib, and dexamethasone (CyBorD) to attempt to salvage his renal function.

While plasmapheresis and CyBorD did achieve some reduction in free light chain ratio burden to 312, renal function did not improve during his admission. He remained in the hospital until outpatient dialysis could be arranged. Unfortunately, due to continued issues with depression, he stopped attending his follow-up appointments and dialysis sessions, resulting in readmission several months later with a similar presentation.

## Discussion

The patient in this case presented with altered mental status secondary to acute kidney failure with anion gap metabolic acidosis and a high osmolar gap. This combination of clinical presentation and laboratory findings (encephalopathy, renal failure with a markedly elevated osmolar gap) is typically associated with toxic ingestion [2,3]. However, the differential for an elevated serum osmolality is broad and includes other etiologies such as shock, renal failure, hyperproteinemia, hyperlipidemia, excessive levels of non-sodium positive ions (e.g. magnesium or lithium), and ketoacidosis, although often to lesser degrees of elevation as seen in toxic alcohols [3,4]. Presenting symptoms of toxic ingestion are often nonspecific, with variability depending on the substance consumed [2-6]. Non-specific features like altered mental status are prominent, as in this case, which can cause difficulty in elucidating further symptom-based differentiation [2,6]. The lack of specificity in clinical symptoms necessitates the consideration of a broad differential [5]. Results of toxic alcohol levels are unlikely to be available in the emergent setting, leaving treatment decisions to be made based on collateral history taking and alternative laboratory evidence [2,5].

One of the key values to consider is the anion gap; elevation is a sign of accumulation of another strong positive cation such as those produced through the metabolism of toxic alcohols or BUN, as in this case [7-9]. An anion gap metabolic acidosis (AGMA) carries with it a distinct differential [10]. Usually, in the absence of other abnormalities, the anion gap in multiple myeloma is decreased or unaffected in cases of IgG or IgM, respectively. In cases of IgA, it can be elevated because its isoelectric point is slightly below physiologic pH, causing it to act as an anion [11]. The AGMA, in this case, was likely influenced by multiple factors, including the patient’s uremia, elevated lactate, gammopathy, and elevated beta-hydroxybutyrate.

The combination of both an AGMA and osmolar gap (the difference between the measured and calculated serum osmolality) may raise suspicion for toxic ingestion, particularly with methanol, ethylene glycol, propylene glycol, or diethylene glycol, as these alcohols accumulate in the serum [5-7,9]. While it is true that elevated osmolar gaps can be found in other causes of acidosis, such as lactic acidosis, ketoacidosis, or renal failure, the gap in those cases is generally less than 20 mOsm/L [3]. An initial anion gap of 26 and an osmolar gap of 42, paired with this patient’s social and psychiatric histories, created a high clinical suspicion for toxic ingestion, prompting appropriate treatment initiation despite ultimately not being the final diagnosis [2,5].

The initial patient presentation, in this case, bore several similarities to that of toxic ingestion, making HD and fomepizole rational choices in the first phases of treatment. However, these measures were unhelpful in treating this patient’s true underlying etiology. This case demonstrates how the osmolar gap can be a beneficial screening tool for toxic ingestion, however, its sensitivity and specificity are limited by a wide range of normal values and the multiple factors that can contribute to its elevation. As such, it must be interpreted with caution [12,13]. Multiple myeloma is known to cause a wide array of pathologies in the kidney and can present in numerous ways, including acute kidney failure and altered mental status [14-16]. AGMA can be present secondary to kidney failure, however, a significant increase in the osmolar gap is not commonly seen [3,16], making this an unusually striking example.

## Conclusions

This case highlights that acute myeloma can present in a dramatic fashion, especially in a healthy individual with a high tolerance for the sequelae of progressive renal failure. While toxic ingestions stereotypically present with high osmolar and anion gaps, in rare cases, these gaps can be caused by a combination of overlapping syndromes such as the hypergammaglobulinemia, uremia, lactic acidosis, and ketoacidosis seen in this case. Notably, pseudohyponatremia, despite a large osmolar gap, may not always be concomitantly present. Identifying profound acidosis with a large osmolar gap as a possible presentation of alternative etiology, such as multiple myeloma, is essential for the preservation of renal function.
